# Supplementary material for: Proteome-wide association study of prostate cancer risk across populations
Source: Nat Commun. 2025 Dec 6;17:3043. doi: 10.1038/s41467-025-66250-5 (PMC13039972; doi:10.1038/s41467-025-66250-5)
Supplement: Supplementary file 1 — Supplementary Information [file 41467_2025_66250_MOESM1_ESM.pdf]

Proteome-wide association study of prostate cancer risk across populations

Supplementary figures

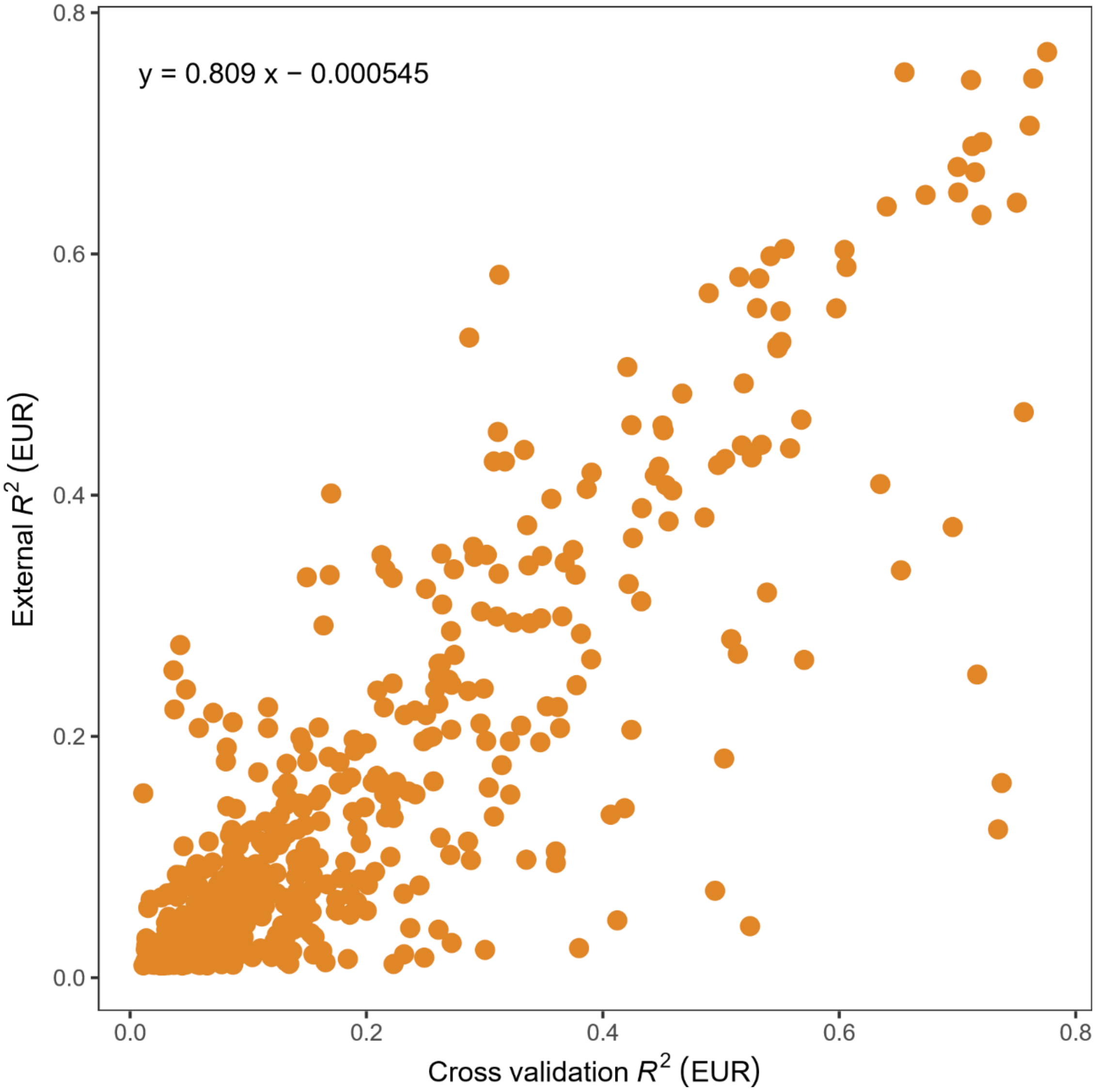

Supplementary Figure 1. Performance of protein genetic prediction models in European ancestry with cross-validation R^2 and external R^2. Sample sizes for Cross validation and External validation are 785 and 1,685, respectively. Source data are provided as a Source Data file.
