# Supplementary material for: Proteome-wide association study of prostate cancer risk across populations
Source: Nat Commun. 2025 Dec 6;17:3043. doi: 10.1038/s41467-025-66250-5 (PMC13039972; doi:10.1038/s41467-025-66250-5)
Supplement: Supplementary file 2 — Description of Additional Supplementary Files [file 41467_2025_66250_MOESM2_ESM.pdf]

Description of Additional Supplementary Files

File Name: Supplementary Data 1

Description: Characteristics of the Established Prediction Models with R^2>0.01.

File Name: Supplementary Data 2

Description: Significant protein-PCa risk associations identified in our study. Associations were tested using the TWAS/FUSION framework (two-sided logistic regression). Multiple test correction was applied for analyses within each population and pan-population meta-analysis.

File Name: Supplementary Data 3

Description: 2ScML robustness analysis of candidate proteins.

File Name: Supplementary Data 4

Description: GO enrichment analysis of genes encoding candidate proteins. GO enrichment analysis was performed using a hypergeometric test (equivalent to a one-sided Fisher's exact test). *P*-values were adjusted for multiple comparisons using the Benjamini-Hochberg method to control the false discovery rate (FDR).

File Name: Supplementary Data 5

Description: Node degree of candidate proteins in PPI network.

File Name: Supplementary Data 6

Description: IPA result of associated proteins. The *p*-values are raw *p*-values from IPA software (one-side).

File Name: Supplementary Data 7

Description: Drug repurposing opportunities of identified proteins.

File Name: Supplementary Data 8

Description: Comparison with reported proteins associated with PCa risk. For all *p*-values shown, two-sided tests were used.
