# Supplementary material for: Proteome-wide association study of prostate cancer risk across populations
Source: Nat Commun. 2025 Dec 6;17:3043. doi: 10.1038/s41467-025-66250-5 (PMC13039972; doi:10.1038/s41467-025-66250-5)
Supplement: Supplementary file 11 — Reporting Summary [file 41467_2025_66250_MOESM11_ESM.pdf]

nature portfolio

Corresponding author(s): Lang Wu

Last updated by author(s): Oct 20, 2025

Reporting Summary

Nature Portfolio wishes to improve the reproducibility of the work that we publish. This form provides structure for consistency and transparency in reporting. For further information on Nature Portfolio policies, see our Editorial Policies and the Editorial Policy Checklist.

Statistics

For all statistical analyses, confirm that the following items are present in the figure legend, table legend, main text, or Methods section.

n/a	Confirmed	
☐	☒	The exact sample size (*n*) for each experimental group/condition, given as a discrete number and unit of measurement
☐	☒	A statement on whether measurements were taken from distinct samples or whether the same sample was measured repeatedly
☐	☒	The statistical test(s) used AND whether they are one- or two-sided<br>*Only common tests should be described solely by name; describe more complex techniques in the Methods section.*
☐	☒	A description of all covariates tested
☐	☒	A description of any assumptions or corrections, such as tests of normality and adjustment for multiple comparisons
☐	☒	A full description of the statistical parameters including central tendency (e.g. means) or other basic estimates (e.g. regression coefficient) AND variation (e.g. standard deviation) or associated estimates of uncertainty (e.g. confidence intervals)
☐	☒	For null hypothesis testing, the test statistic (e.g. *F*, *t*, *r*) with confidence intervals, effect sizes, degrees of freedom and *P* value noted<br>*Give P values as exact values whenever suitable.*
☐	☒	For Bayesian analysis, information on the choice of priors and Markov chain Monte Carlo settings
☐	☒	For hierarchical and complex designs, identification of the appropriate level for tests and full reporting of outcomes
☐	☒	Estimates of effect sizes (e.g. Cohen's *d*, Pearson's *r*), indicating how they were calculated

Our web collection on statistics for biologists contains articles on many of the points above.

Software and code

Policy information about availability of computer code

Data collection	The Multi-Ethnic Study of Atherosclerosis (MESA) included approximately 6,500 men and women without known clinical cardiovascular disease at baseline, aged 45–84 years who were initially enrolled in 2000 77. Baseline information and blood samples of the MESA participants were collected at their initial visit, which took place in six US states (New York, Maryland, Illinois, California, Minnesota, and North Carolina). Detailed information on the MESA study design can be found elsewhere
Data analysis	Genotype Data Processing and Quality Control<br>The genotype data utilized in this study was generated using Affymetrix SNP 6.0, which was obtained from the MESA SNP Health Association Resource (SHARe) study (phs000420.v6.p3) and imputed on the Michigan imputation server (Minimac4.v1.0.0) using the 1000 Genomes reference panel 78. For each population of interest, we excluded subjects who were identified as related via identity-by-descent (IBD) analysis, employing an in-house script that considered independent (R2 < 0.2) and common (minor allele frequency (MAF) ≥ 5%) SNPs. This process resulted in a final sample of 1,971 unrelated male subjects, including 450 of African background, 758 of European background, 289 of Asian background, and of 474 Hispanic/Latino background. Subsequently, SNPs were filtered based on pre-determined criteria including MAF > 0.05, genotyping missingness < 5%, and adherence to Hardy-Weinberg equilibrium (HWE, P > 5×10-6) using PLINK v1.9 software. After filtering for variants present in the 1000 Genomes reference panel, a total of 8,011,863, 5,814,519, 5,308,357, and 6,058,753 high-quality SNPs were retained for African, European, Asian, and Hispanic/Latino populations, respectively. The LD-pruned (R2 < 0.2), common (MAF ≥ 5%), and genotyped variants within 200 base pair windows were used to calculate genetic principal components (PCs) using the EIGENSOFT software 79.<br><br>Proteomic Data Processing<br>Proteomic profiling was conducted using the aptamer-based SomaScan assay, which quantified 7,289 human proteins. We excluded 10 SOMAmers whose target protein-encoding genes lacked positional information in the BioMart 80 database. An additional 280 SOMAmers

targeting proteins encoded on sex chromosomes were removed to focus the analysis on plasma proteins or protein complexes encoded by autosomal genes. After these exclusions, 6,999 proteins were retained for downstream analysis. The residuals of 6,999 protein abundances after adjusting for covariates were transformed using a rank-based inverse normal transformation for model building.

Building Protein Genetic Prediction Models

To identify informative SNPs for model building, we first conducted pQTL analyses adjusting for study site, BMI, sex, age, cigarette smoking status, pack-years of cigarette smoking, and top ten PCs. Significant cis-pQTLs were defined as SNPs within cis-regions associated with a protein at FDR < 0.05, while significant trans-pQTLs were defined as SNPs in trans-regions with P < 5×10-9. These significance thresholds were chosen to maximize the inclusion of potentially informative SNPs while minimizing excess noise 81. We further extracted non-strand-ambiguous SNPs within 100kb of significant cis- and trans-pQTLs to serve as candidate predictors for each protein.

We used TWAS/FUSION framework 82 to construct subsequent genetic prediction models. Four methods were used for model construction: best linear unbiased predictor (BLUP), least absolute shrinkage and selection operator (LASSO), elastic net, and top SNPs (top1). BLUP estimates the joint effect sizes of all SNPs using a single variance component 83. LASSO is a penalized regression method utilizing L1 regularization techniques to produce sparse models 84. As a generalization of the LASSO, elastic net linearly combines the L1-penalty of LASSO and L2-penalty of ridge regression, to select highly correlated variables together 85. For each protein of interest, the prediction model with the most significant cross-validation P-value was retained. Only models with a cross-validation R2 > 0.01 (indicating they explain more than 1% of the variance, corresponding to a minimum ~10% correlation between predicted and measured protein levels) were included in subsequent association analysis. This threshold is commonly applied in similar studies 8,81,86–89. Cross-validation was performed using a five-fold scheme, where the dataset was randomly divided into five equal parts. In each fold, models were trained on 80% of the data and tested on the remaining 20%, rotating such that each subset served as a test set once. The final cross-validation performance was calculated by regressing observed protein levels against the predicted values aggregated from all test folds. This adjusted R2 accounts for model complexity and sample size, providing a conservative measure of the variance explained by the genetic predictors.

We compared R2 and H^2 across African, European, Asian, and Hispanic/Latino populations using the Kruskal–Wallis test, given the non-normal distribution of R2 and H^2 values. When significant, post hoc pairwise comparisons were performed using Dunn's test with Bonferroni correction. Analyses were conducted in R (version 4.1.2) using the 'FSA' package.

For manuscripts utilizing custom algorithms or software that are central to the research but not yet described in published literature, software must be made available to editors and reviewers. We strongly encourage code deposition in a community repository (e.g. GitHub). See the Nature Portfolio guidelines for submitting code & software for further information.

Data

Policy information about availability of data

All manuscripts must include a data availability statement. This statement should provide the following information, where applicable:

- Accession codes, unique identifiers, or web links for publicly available datasets
- A description of any restrictions on data availability
- For clinical datasets or third party data, please ensure that the statement adheres to our policy

Specific genome, proteome, and covariate data of MESA 77 have been deposited to the database of Genotypes and Phenotypes (dbGaP) under accession code phs000209.v13.p3 (https://www.ncbi.nlm.nih.gov/projects/gap/cgi-bin/study.cgi?study_id=phs000209.v13.p3). Additional data of MESA are available through a concept proposal application via MESA Genetics P and P Committee. These data are available under restricted access to protect participant privacy and comply with informed consent agreements. For data available through dbGaP, access is limited to qualified researchers who submit a Data Access Request (DAR) through dbGaP. Such requests must include a research use statement and a data use certification signed by the principal investigator and the institutional signing official. Requests will be reviewed by the relevant Data Access Committee (DAC) with a timeline developed by dbGaP. Once approved by dbGaP, access will be granted for one year. It requires an annual renewal via dbGaP to continue access beyond the initial 12-month period. Individual level data of genotype and proteomic data of INTERVAL 31 study are available under controlled access in European Genome-phenome Archive (EGA) under accession number EGAS00001002555 (https://ega-archive.org/studies/EGAS00001002555). Access is restricted to protect participant privacy and is granted to qualified researchers following approval by the EGA Data Access Committee. Researchers interested in accessing these data can submit a request via the EGA data access portal, and access can be provided after the EGA Data Access Committee reviews and approves it. DAC Aim to respond to all initial requests in less than 2 weeks. The length of time you can access the data depends on the terms set by the DAC. The publicly available summary statistics of multi-population PCa GWAS 4 are available on the GWAS Catalog (https://www.ebi.ac.uk/gwas/). These statistics are categorized by different racial/ethnicity groups with the following accession codes: European (GCST90274714, https://www.ebi.ac.uk/gwas/studies/GCST90274714) 4, African (GCST90274715, https://www.ebi.ac.uk/gwas/studies/GCST90274715) 4, Asian (GCST90274716, https://www.ebi.ac.uk/gwas/studies/GCST90274716) 4, and Hispanic/Latino (GCST90274717, https://www.ebi.ac.uk/gwas/studies/GCST90274717) 4. The remaining data are available within the Article, Supplementary Information or Source Data file.

Research involving human participants, their data, or biological material

Policy information about studies with human participants or human data. See also policy information about sex, gender (identity/presentation), and sexual orientation and race, ethnicity and racism.

Reporting on sex and gender	In In this study, we focused on 2,013 male subjects who had no self-reported cancer diagnosis and no ICD-9 or ICD-10 cancer diagnoses in their hospitalization records or death certificates at baseline, including 453 African, 765 European, 296 Asian, and 499 Hispanic/Latino individuals residing in the USA. Population groups in our analysis were initially defined based on self-reported race/ethnicity as recorded in the original studies.
Reporting on race, ethnicity, or other socially relevant groupings	In this study, we focused on 2,013 male subjects who had no self-reported cancer diagnosis and no ICD-9 or ICD-10 cancer diagnoses in their hospitalization records or death certificates at baseline, including 453 African, 765 European, 296 Asian, and 499 Hispanic/Latino individuals residing in the USA. Population groups in our analysis were initially defined based on self-reported race/ethnicity as recorded in the original studies.
Population characteristics	In this study, we focused on 2,013 male subjects who had no self-reported cancer diagnosis and no ICD-9 or ICD-10 cancer diagnoses in their hospitalization records or death certificates at baseline, including 453 African, 765 European, 296 Asian,

	and 499 Hispanic/Latino individuals residing in the USA. Population groups in our analysis were initially defined based on self-reported race/ethnicity as recorded in the original studies. All subjects had data available on blood protein levels (v1), genotype, and relevant covariates, including body mass index (BMI), sex, age, cigarette smoking status, and pack-years of cigarette smoking.
Recruitment	The Multi-Ethnic Study of Atherosclerosis (MESA) included approximately 6,500 men and women without known clinical cardiovascular disease at baseline, aged 45–84 years who were initially enrolled in 2000 77. Baseline information and blood samples of the MESA participants were collected at their initial visit, which took place in six US states (New York, Maryland, Illinois, California, Minnesota, and North Carolina). Detailed information on the MESA study design can be found elsewhere 77. The MESA study protocol was reviewed and approved by the Institutional Review Boards (IRBs) of all participating institutions as well as by the National Heart, Lung, and Blood Institute (NHLBI). All participants provided written informed consent to participate in the parent study and received financial compensation.
Ethics oversight	. The MESA study protocol was reviewed and approved by the Institutional Review Boards (IRBs) of all participating institutions as well as by the National Heart, Lung, and Blood Institute (NHLBI).

Note that full information on the approval of the study protocol must also be provided in the manuscript.

Field-specific reporting

Please select the one below that is the best fit for your research. If you are not sure, read the appropriate sections before making your selection.

[x] Life sciences   [ ] Behavioural & social sciences   [ ] Ecological, evolutionary & environmental sciences

For a reference copy of the document with all sections, see nature.com/documents/nr-reporting-summary-flat.pdf

Life sciences study design

All studies must disclose on these points even when the disclosure is negative.

Sample size	This study includes 453 African, 765 European, 296 Asian, and 499 Hispanic/Latino individuals residing in the USA and 6,999 proteins data.
Data exclusions	For each population of interest, we excluded subjects who were identified as related via identity-by-descent (IBD) analysis, employing an in-house script that considered independent (R2 < 0.2) and common (minor allele frequency (MAF) ≥ 5%) SNPs. This process resulted in a final sample of 1,971 unrelated male subjects, including 450 of African background, 758 of European background, 289 of Asian background, and of 474 Hispanic/Latino background.
Replication	We conducted external validation of the European population models using data from 1,685 healthy European males of the INTERVAL study, which measured plasma concentrations of 3,622 proteins.
Randomization	This study is based on data from the Multi-Ethnic Study of Atherosclerosis (MESA), a prospective cohort study. Randomization was not applicable, as participants were recruited using population-based sampling strategies rather than randomized allocation
Blinding	MESA is an observational cohort study without experimental intervention; therefore, participant blinding was not applicable. However, technicians and readers performing imaging and laboratory assessments were blinded to participants' clinical characteristics, and data analysts worked with de-identified datasets.

Reporting for specific materials, systems and methods

We require information from authors about some types of materials, experimental systems and methods used in many studies. Here, indicate whether each material, system or method listed is relevant to your study. If you are not sure if a list item applies to your research, read the appropriate section before selecting a response.

Materials & experimental systems

n/a	Involved in the study	
[x]	[ ]	Antibodies
[x]	[ ]	Eukaryotic cell lines
[x]	[ ]	Palaeontology and archaeology
[x]	[ ]	Animals and other organisms
[x]	[ ]	Clinical data
[x]	[ ]	Dual use research of concern
[x]	[ ]	Plants

Methods

n/a	Involved in the study	
[x]	[ ]	ChIP-seq
[x]	[ ]	Flow cytometry
[x]	[ ]	MRI-based neuroimaging

Plants

Seed stocks	*Report on the source of all seed stocks or other plant material used. If applicable, state the seed stock centre and catalogue number. If plant specimens were collected from the field, describe the collection location, date and sampling procedures.*
Novel plant genotypes	*Describe the methods by which all novel plant genotypes were produced. This includes those generated by transgenic approaches, gene editing, chemical/radiation-based mutagenesis and hybridization. For transgenic lines, describe the transformation method, the number of independent lines analyzed and the generation upon which experiments were performed. For gene-edited lines, describe the editor used, the endogenous sequence targeted for editing, the targeting guide RNA sequence (if applicable) and how the editor was applied.*
Authentication	*Describe any authentication procedures for each seed stock used or novel genotype generated. Describe any experiments used to assess the effect of a mutation and, where applicable, how potential secondary effects (e.g. second site T-DNA insertions, mosiacism, off-target gene editing) were examined.*
